# Carbon, Nitrogen, and Phosphorus Stoichiometry and Plant Growth Strategy as Related to Land-Use in Hangzhou Bay Coastal Wetland, China

**DOI:** 10.3389/fpls.2022.946949

**Published:** 2022-07-06

**Authors:** Jing Xiong, Xuexin Shao, Haijing Yuan, Enjun Liu, Ming Wu

**Affiliations:** ^1^Research Institute of Subtropical Forestry, Chinese Academy of Forestry, Hangzhou, China; ^2^College of Landscape Architecture, Nanjing Forestry University, Nanjing, China; ^3^Ningbo Wetlands Research Center, Ningbo, China

**Keywords:** land-use, coastal wetlands, stoichiometry, nutrient stocks, plant strategy, wetland reclamation, plant invasion

## Abstract

Ecological stoichiometry can not only instruct soil nutrient stocks and availability, but also indicated plant growth strategy and adaptability to environmental changes or stress. This study was carried out to examine the plant–soil Carbon (C), Nitrogen (N), and Phosphorus (P) stoichiometry distributions and patterns in three tidal wetlands [mudflat (MF), native *Phragmites australis*-dominated community wetland (NW), invasive *Spartina alterniflora*-dominated community wetland (IW)], and one reclaimed *P. australis*-dominated community wetland (RW) in Hangzhou Bay coastal wetland. The results showed that land-uses have more effect on C and N contents, and C:N and N:P ratios in plant than in soil, P content and C:P ratios more affected by plant organ and soil depth. Compared to land-use, both plant organ and soil depth have stronger effects on C, N, and P stoichiometry. Among tidal wetlands, plant N content and C:P, N:P ratios were significantly higher in NW than in IW. In contrast, plant C, N, and P contents and C:P and N:P ratios were significantly lower in RW, and plant C:N was higher. Soil C, N, and P stocks were similar between tidal wetlands, and were significant higher than those of RW, indicating that reclamation were not beneficial to soil nutrient storage. In the NW, soil N availability was relatively high, and P availability was relatively low; and leaf N:P was 15.33, which means vegetation was co-limited by N and P nutrients. In addition, plants in the NW mainly adopted a conservative growth strategy, with a significantly low aboveground biomass of 1469.35 g·m^2^. In the RW, soil N availability was relatively low, P availability was relatively high, and leaf N:P was 3, which means vegetation was limited by N nutrient. In addition, plants in the RW mainly adopted a rapid growth strategy, with a significantly high aboveground biomass of 3261.70 g·m^2^. In the IW, soil N availability was relatively low, soil P availability was relatively high, and leaf N:P was 5.13, which means vegetation was limited by N nutrient. The growth strategy and aboveground biomass (2293.67 g·m^2^) of the IW were between those of the NW and RW. Our results provide a reference for nutrient management and evaluating the impacts of land-use types on coastal wetland ecosystems.

## Introduction

Ecological stoichiometry focus on the balance of energy and chemical elements in an ecosystem; and provide a powerful tool for understanding nutrient biogeochemistry and ecological process at individual and ecosystem levels (Reich and Oleksyn, 2004; Elser et al., 2007). Carbon (C), nitrogen (N), and phosphorus (P) are abundant and essential elements for plants and ecosystems (Luo et al., 2020; Hui et al., 2021). Their availabilities and stoichiometric ratios of C, N, and P can significantly affect plant growth and community composition (Högberg et al., 2017; Urbina et al., 2017) and can indicate the nutrient dynamics and limitation of vegetation under changing conditions (Güsewell, 2004; Reich and Oleksyn, 2004; Xiong et al., 2021). Changes in the contents and pools of C, N, and P in soil may alter the C, N, and P ratios of various ecosystem components, thereby affecting the structure and function of an ecosystem (Wang et al., 2019; Crovo et al., 2021). In addition, the C:N, C:P, and N:P ratios of soil are also important indicators of soil quality and nutrient supply capacity. Therefore, these parameters can provide theoretical guidance for managing soil nutrients, and help us understand the response of element changes to global environmental changes and carbon cycle processes (Zhang et al., 2015; Chen and Chen, 2021; Zheng et al., 2021). Compared with the relatively stable C, N, and P stoichiometry of plants, those of soil are more variable (Bui and Henderson, 2013). Therefore, it is helpful to interpret the corresponding ecological effects to study the distribution characteristics of the C, N, and P contents and ratios of different ecosystems.

Changes in land-use caused by natural and human interferences (e.g., forest conversion, farming, and plant invasion) can significantly affect the ecological stoichiometry of C, N, and P both in plants and soils (Wang et al., 2019; Luo et al., 2020; Crovo et al., 2021; Zheng et al., 2021; Tang et al., 2022). C, N, and P stoichiometry of the top soil was more sensitive to land-use (e.g., woodland, upland, and paddy; Tang et al., 2022). Compared to the woodland, soil C was decreased and P was increased of upland agriculture, while soil C, N, and P content were all increased of paddy (Zheng et al., 2021). Land-use changed the competitive relationships of plant through change the soil C, N, P, and K stoichiometry (Wang et al., 2014a). The invasive success of *Spartina alterniflora* may decrease the ecosystem N:P ratio by change the soil N and P capacity and future adjust below- and above-ground trophic chains (Wang et al., 2019). In summary, land-use can result in stoichiometry imbalance and a influence both in soil and plant productivity.

Coastal wetlands have extremely high biodiversity and productivity, and play vitally important functions that cannot be replaced by other ecosystems (Hu et al., 2021). However, global changes, such as reclamation or plant invasion due to human activity, have already change the land-use and degraded and damaged ecosystem functions (Ma et al., 2014). However, how the plant–soil C, N, and P stoichiometry is affected by these changes in coastal wetland remains unknown. The coastal wetland of Hangzhou Bay is the most prominent area for artificial reclamation and utilization; the ecosystem in this region has become extremely unstable and fragile (Yang et al., 2004; Feng and Bao, 2006). Therefore, it is of great significance to explore how different land-use types in this area affect the ecological stoichiometry of plants and soil. Accordingly, this study aims to: (i) investigate the distributions of stoichiometry of C, N, and P in plant organs (leaf, stem, and root) and soil (depths of 0–10, 10–30, 30–60, and 60–100 cm), and (ii) explore their relationships with the environmental conditions of different land-use types (mudflat, native wetland, reclaimed wetland, and invasive wetland). Therefore, we test the following hypotheses: (i) the C, N, and P contents and ratios of plants and soil vary between land-uses, (ii) reclamation and plant invasion are not beneficial soil nutrient stocks, and (iii) land-use can affect the plant growth strategy by changing ecological stoichiometry and habitat.

## Materials and Methods

### Study Area

Hangzhou Bay is a trumpet-shaped tidal estuary located along the north–south demarcation line of coastal wetlands in China. The bay is one of the most abundant areas of waterfowl in eastern China in winter, and is also an important station on the migration route of migratory birds from East Asia to Australasia. The total estimated value of ecosystem services in the southern coastal wetlands of Hangzhou Bay is approximately 1127.83 × 10^8^ Yuan (Ning et al., 2017). This study area is in the southern part of Hangzhou Bay (30°10′ N–30°42′ N, 120°55′ E–121°30′ E), and belongs to the northern subtropical maritime monsoon climate zone, with four distinct seasons. The area has a mean annual temperature of 16°C, a mean annual precipitation of 1,273 mm, an annual sunshine duration of 2,038 h, an annual frost-free period of 244 days, and irregular semi-diurnal tides. The soil type is the littoral salinity subtype.

**Figure 1:**
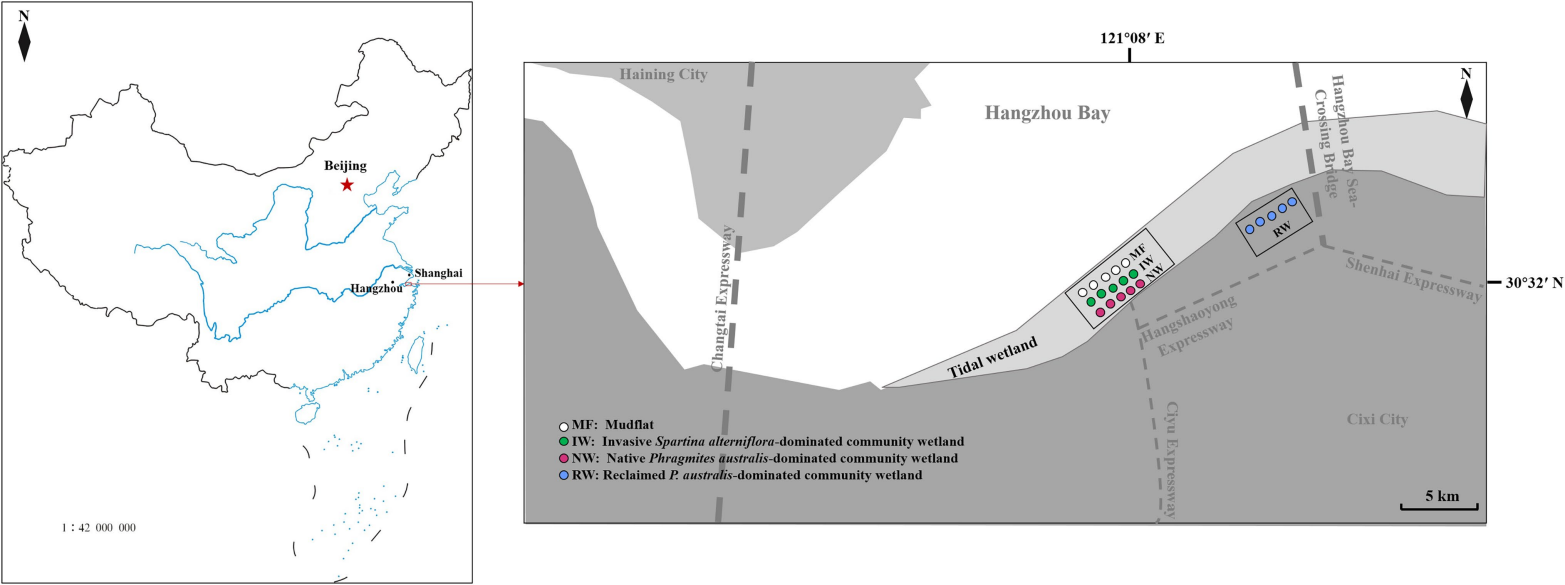
The location of the study area. MF, mudflat; NW, native *Phragmites australis*-dominated community wetland; IW, invasive *Spartina alterniflora*-dominated community wetland; and RW, reclaimed *P. australis*-dominated community wetland.

Reclamation activities and plant invasion are two major ecological issues affecting the “blue carbon” balance. Reclamation activities can lead to habitat destruction in coastal wetlands, while in turn can affect ecosystem health and even lead to habitat loss. *Spartina alterniflora* invaded and rapidly occupied the ecological niche of native plants, resulting in changes in plant communities and affecting the status of invasive ecosystems. Both anthropogenic reclamation and plant invasion have changed the land-use, and may have vital impacts on the balance and cycling of C, N, and P in coastal wetland ecosystems. And mudflat and native plant-dominated community wetland were selected as control. Therefore, mudflat (MF) and native *Phragmites australis*-dominated community wetland (NW), and reclaimed *P. australis*-dominated community wetland (RW), and invasive *S. alterniflora*-dominated community wetland (IW) were selected in the coastal wetland of the southern part of Hangzhou Bay (Table 1; Figure 1). The MF is in a low-tide flat area with no plants or human activity. The NW is in the middle-high tide flat area, has a single native *P. australis* community, and is unaffected by human management or disturbance. The RW is in the seawall area and is unaffected by the tide and human management. This area was restored and regenerated with a single native *P. australis* community after it was reclaimed around 2017. The IW is in a low-tidal flat area with a single invasive *S. alterniflora* community. *Spartina alterniflora* (16 m^2^) was first planted in the mudflat area of Yuhuan County in Zhejiang Province, China, in 1983. This species, then expanded rapidly and naturally, with its invasive area reaching 5,092 hm^2^ in the eastern coastal area (Zhang, 2010).

**Table 1:** Description of four land-use types in the coastal wetland area of Hangzhou Bay.

Land use	Tidal effect	Longitude and latitude	Mean annual temperature	Mean annual precipitation	Plant type	History and management of land use
Mudflat (MF)	Yes	30.37 N, 121.08 E	16°C	1,273 mm	No plants	Unmanaged
Native *Phragmites australis*-dominated community wetland (NW)		30.32 N, 121.08 E			*Phragmites australis*	Native plants, without human management or disturbance
Invasive *Spartina alterniflora*-dominated community wetland (IW)		30.32 N, 121.08 E			*Spartina alterniflora*	*Spartina alterniflora* rapidly invaded the low-tidal flat area after it was introduction by humans in 1983 and subsequently invaded the study area around 2016
Reclaimed *Phragmites australis*-dominated community wetland (RW)	No	30.36 N, 121.13 E			*Phragmites australis*	Natural restoration occurred after reclamation around 2017, after which there was no tidal flat, human management, or human disturbance

### Field Sampling and Analysis

Plant and soil samples were collected from the MF, NW, RW, and IW in mid-July 2021. At each land-use type, five 20 m × 20 m sample sites were studied. Each sample site was more than 200 m apart, avoiding tidal gullies and edge zones, with large vegetation areas and similar growth status in vegetated samples. A 1 m × 1 m sample plot was randomly selected in each sample site to investigate the vegetation density, height, diameter, and aboveground biomass (Supplementary Table S1). Twelve healthy plants were randomly selected in each 1 m × 1 m sample plot, and their root, stem, and leaf were separated and dried to determine their C, N, and P contents, respectively.

In four land use types, the soil physiochemical properties of soil profile were investigated. The temperature was measured by a thermometer; soil moisture was measured by the weighing method; pH and salinity were measured by the multi-functional pH meter and conductivity meter, respectively (Supplementary Table S2). According to the response of soil depth to the land-use, root distribution characteristics and soil oxygen status, the soil samples were divided into four layers (Angle et al., 2017; Tang et al., 2022). Five to six disturbed soil samples were collected in each sample plot using a soil drill from each soil depth (0–10, 10–30, 30–60, and 60–100 cm) and combined to form a composite soil sample. The soil C, N, and P stocks in soil layers were determined by the following equations (Tang et al., 2022):


(1) $$\mathrm{Soil}\;C\;\mathrm{stock}\;\left(\mathrm{Cs}\right)\;\left(\mathrm{Mg}\cdot {\mathrm{ha}}^{-1}\right)=\frac{C\times \mathrm{Bulk density}\times \mathrm{soil depth}}{10}$$

(2) $$\mathrm{Soil}\;N\;\mathrm{stock}\;\left(\mathrm{Ns}\right)\;\left(\mathrm{Mg}\cdot {\mathrm{ha}}^{-1}\right)=\frac{N\times \mathrm{Bulk density}\times \mathrm{soil depth}}{10}$$

(3) $$\mathrm{Soil}\;P\;\mathrm{stock}\;\left(\mathrm{Ps}\right)\;\left(\mathrm{Mg}\cdot {\mathrm{ha}}^{-1}\right)=\frac{P\times \mathrm{Bulk density}\times \mathrm{soil depth}}{10}$$

where C is soil C content (g·kg^−1^); N is soil N content (g·kg^−1^); P is soil P content (g·kg^−1^). Total soil C, N, and P stocks within the 100 cm depths were weighed and summed for all four soil layers (0–10, 10–30, 30–60, and 60–100 cm).

The C and N contents of plant organs and soil were determined by the elemental analyzer, and P content of plant organs and soil were determined by a digestion procedure with HNO_3_–HF–HClO_4_ (Jackson, 1958).

### Calculation and Statistical Analysis

We log10-transformed all the data before statistical analyses to meet normality of variance requirements. Nested ANOVA were used to examine the effects of three plant organs under three land-use types on the C, N, and P stoichiometry. Nested analysis of variance were used to examine the effects of four soil depths under four land-use types on the C, N, and P stoichiometry. One-way ANOVA were performed to test the effects of three land-use types (NW, RW, IW, and tidal wetlands) with three plant organs on the C, N, and P contents and ratios in plant. And One-way ANOVA were also performed to test the effects of four land-use types (MF, NW, RW, IW, and tidal wetlands) with four soil depths on the C, N, and P contents and ratios. These ANOVA were carried out using the IBM SPSS Statistics 22 software (SPSS. Inc., Chicago, IL, United States). Pearson’s correlation analysis was used to explore the relationships between C, N, and P contents, ratios and stocks of plant organs and soil depths. Principal component analysis (PCA) was used to explore the relationships between C, N, and P stoichiometry and plant growth traits, and soil physiochemical properties under four land-use types. Aggregated boosted tree analysis (ABT) and random forest analysis were used to explore the key factors of soil C, N, and P stocks and plant aboveground biomass. The PCA was conducted using the ggvegan package, Pearson’s correlation analysis was conducted using the psych and tidyverse package, the ABT was conducted using the dismo package, and random forest analysis was conducted using the randomForest package in R software (Version 4.1.3), respectively.

## Results

### Distribution of Plant Organs C, N, and P Contents and Ratios of Three Vegetated Wetlands

Land-uses significantly affected plant C and N contents and plant C:N and N:P ratios (*p* < 0.05), plant organs in the same land-use just did not significantly affected plant C content (*p* < 0.05; Table 2). Compared to the RW, the plant C and N contents, and C:P ratios of tidal wetlands were significantly higher, while the C:N ratio was significantly lower (*p* < 0.05; Supplementary Figures S1 A,B,D,E). In addition, there was a large difference in plant C, N, and P contents and ratios between RW and NW. At the same time, there were relatively small differences between RW and IW (*p* < 0.05; Supplementary Figure S1).

**Table 2:** Nested ANOVA results for the effects of different land-use types, plant organs, and soil depths and on the C, N, and P contents and ratios, and C, N, and P stocks.

	DF	Plant C content	Plant N content	Plant P content	Plant C:N ratio	Plant C:P ratio	Plant N:P ratio			
Land-use	2	29.696^**^	63.266^***^	0.110^ns^	9.209^*^	1.060^ns^	11.303^**^			
Plant organ (Land-use)	6	1.850^ns^	7.611^***^	28.048^***^	21.865^***^	22.884^***^	22.423^***^			
	**DF**	**Soil C content**	**Soil N content**	**Soil P content**	**Soil C:N ratio**	**Soil C:P ratio**	**Soil N:P ratio**	**Soil C stock**	**Soil N stock**	**Soil P stock**
Land-use	3	0.762^ns^	5.737^*^	1.099^ns^	12.545^**^	0.811^ns^	3.120^ns^	3.400^ns^	2.245^ns^	0.200^ns^
Soil depth (Land-use)	12	32.894^***^	15.344^***^	1.180^ns^	5.860^***^	4.150^**^	3.379^**^	1.640^ns^	22.056^***^	27.581^***^

DF, degree of freedom for each test. ns, nonsignificant.

*** *p* < 0.001;

** *p* < 0.01;

* *p* < 0.05.

**Figure 2:**
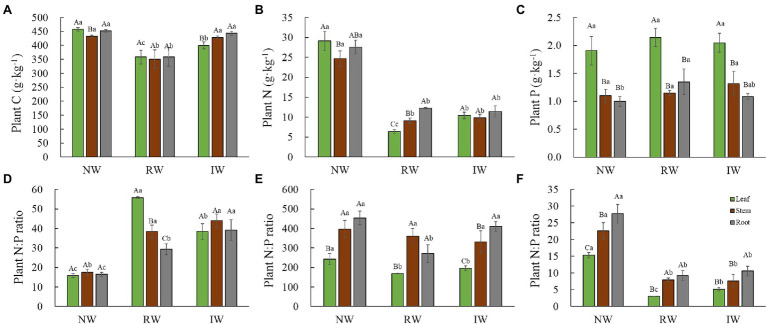
Distributions of the plant Carbon (C) content **(A)**, plant N content **(B)**, plant P contents **(C)**, plant C:N ratio **(D)**, plant C:P ratio **(E)**, and plant N:P ratio (**F**; means ± SD, *n* = 3) under different land-use types in Hangzhou Bay. Different capital letters for the same land-use type indicate a significant difference among plant organs at *p* < 0.05, and different lower-case letters for the same organ indicate a significant difference among land-use types at *p* < 0.05.

The patterns of P content and C:P and N:P among plant organs were contrasting and were not affected by land-use type (Figures 2C,E,F). The leaf P content was significantly higher, while the leaf C:P and N:P ratio were significantly lower than those in root and stem (*p* < 0.05). However, land-use type affected the patterns of C and N contents and C:N ratio among plant organs (Figures 2A,B,D). Compared to the NW, leaf N allocation was significantly decreased; and root N allocation was significantly increased, while the C:N ratio was higher in leaf than root in RW. In addition, the leaf C allocation was significantly decreased; the stem N allocation were significantly increased in the IW (*p* < 0.05).

### Distribution of Soil C, N, and P Contents and Ratios in Different Land-Use Types

Land-uses just significantly affected soil N content and soil C:N ratios (*p* < 0.05), soil depths in the same land-use just did not significantly affected soil P content (*p* > 0.05; Table 2). In general, soil C and N contents, and C:P and N:P ratios in tidal wetlands, especially in IW, were significantly higher than those in the RW, while soil C:N was significantly lower (*p* < 0.05; Supplementary Figures S2 A,B,D,E).

**Figure 3:**
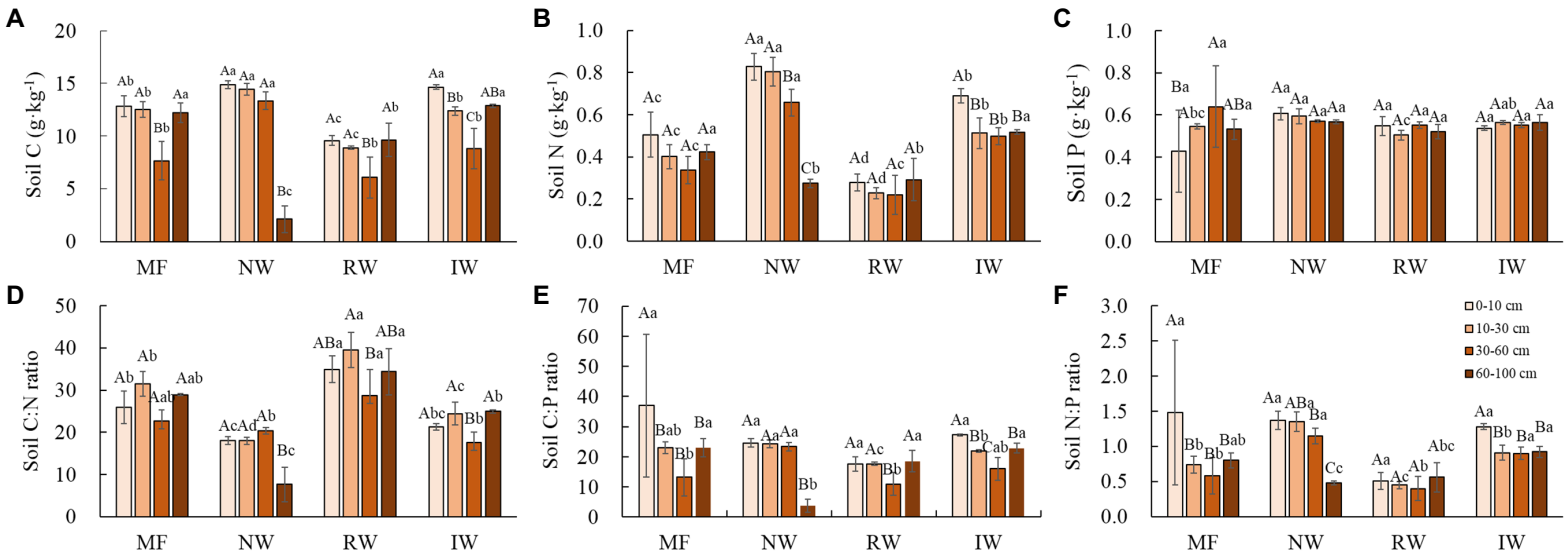
Distributions of the soil C content **(A)**, soil N content **(B)**, soil P contents **(C)**, soil C:N ratio **(D)**, soil C:P ratio **(E)**, and soil N:P ratio (**F**; means ± SD, *n* = 3) under different land-use types in Hangzhou Bay. Different capital letters in the same land-use type indicate a significant difference among soil depths at *p* < 0.05, and different lower-case letters in the same soil depth indicate a significant difference among land-use types at *p* < 0.05.

Land use type also affected the distribution characteristics and spatial patterns of soil C and N contents and C:N, C:P, and N:P ratios, but had little influence on soil P content (*p* > 0.05; Figures 3A–F). In the MF, soil C and N contents, C:P and N:P ratios first decreased and then decreased with increasing soil depth, while the soil P content and C:N ratio showed the opposite trend. In the NW, the soil C and N contents, and C:N, C:P, and N:P ratios at the 60–100 cm were significantly lower than those at other soil depths (*p* < 0.05). In the RW, the soil C content, and C:N and C:P at the 30–60 cm were significantly lower than those in other soil depths (*p* < 0.05). In the IW, the soil C content, C:N, and C:P ratios first increased and then decreased with the increasing soil depth, and the soil N and N:P ratio at the 0–10 cm depth were significantly higher than those at other soil depths (*p* < 0.05).

In terms of spatial patterns, at the 0–10 and 10–30 cm depth, the soil C and N contents significantly decreased in this order: NW > IW > MF > RW (Figures 3A,B). At the 0–10 cm depth, the soil C:N ratio was significantly lower in the NW and significantly higher in the RW (*p* < 0.05; Figure 3D). At the 10–30 cm depth, the highest soil C:P ratio and lowest soil C:N ratio were observed in the NW, the highest soil C:N and lowest soil C:P and N:P ratios were observed in the RW, and soil N:P ratio was significantly larger in MF (*p* < 0.05; Figures 3D–F). At the 30–60 cm depth, soil C and N contents in the NW were the largest, followed by IW, and MF and RW were the smallest (Figures 3A,B). Soil C:P and N:P ratios of the NW and IW were significantly higher than those in MF and RW, while the soil C:N ratio was the opposite (*p* < 0.05; Figures 3D–F). At the 60–100 cm depth, the largest soil C and N contents, C:N, C:P, and N:P ratios were observed in the MF and IW, followed by the RW, and were lowest in the NW (Figures 3A,B,D–F).

### Distributions of Soil C, N, and P Stocks in Different Land-Use Types

**Figure 4:**
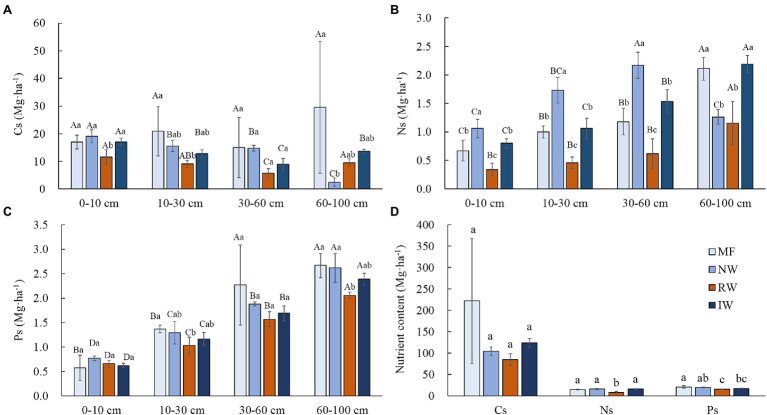
Distributions of the soil C stock **(A)**, soil N stock **(B)**, soil P stock **(C)**, and total soil nutrient stock (**D**; means ± SD, *n* = 3) under different land-use types in Hangzhou Bay. Cs, soil C stock; Ns, soil N stock; and Ps, soil P stock. Different capital letters in the same wetland type indicate a significant difference among soil depths at *p* < 0.05, and different lower-case letters in the same soil depth indicate a significant difference among wetland types at *p* < 0.05.

Land-uses did not significantly affected Cs, Ns, and Ps (*p* > 0.05), while soil depths in the same land-use did had extremely significant effects on Ns, and Ps (*p* < 0.001; Table 2). In general, Cs, Ns, and Ps of each soil depth were lower or significantly lower in RW than tidal wetland (*p* < 0.05). Cs decreased with the increasing soil depth from 0 to 60 cm, and then varied with land-use types at the 60–10 cm depth (Figure 4A). Ns and Ps increased with the increasing soil depth from 0 to 100 cm (Figures 4B,C). There was no significant difference in total Cs (*p* > 0.05), total Ns and Ps were significantly lower in the RW than tidal wetland (*p* < 0.05; Figure 4D).

### Relationships Between C, N, and P Stoichiometry and Stocks and Habitant Conditions

**Figure 5:**
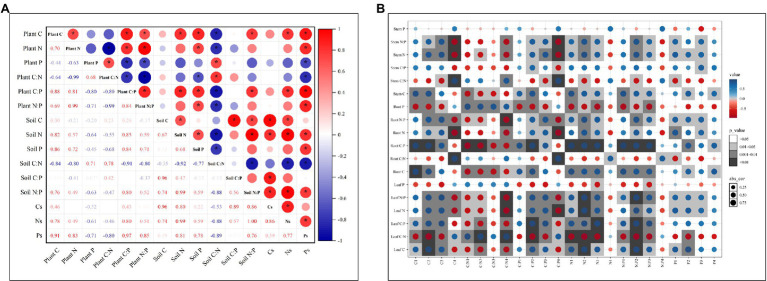
Correlation analysis between C, N, and P contents and ratios of plants and soils **(A)**, and between C, N, P contents and ratios of plant organs and soil depths **(B)**. Cs, soil C stock; Ns, soil N stock; and Ps, soil P stock. C1, N1, P1, C:N1, C:P1, and N:P1 are the soil C, N, and P contents and ratios in 0–10 cm depth. C2, N2, P2, C:N2, C:P2, and N:P2 are the soil C, N, and P contents and ratios in 10–30 cm depth. C3, N3, P3, C:N3, C:P3, and N:P3 are the soil C, N, and P contents and ratios in 30–60 cm depth. C4, N4, P4, C:N4, C:P4, and N:P4 are the soil C, N, and P contents and ratios in 60–100 cm depth.

Pearson’s analysis showed that C, N, and P contents and ratios in plant or soil all have strong self-correlation. Plant C, N, and P contents and ratios were more likely correlated with soil P content, C:N ratio and Ps (*p* < 0.05; Figure 5A). Soil C, N, and P contents and ratios in each soil depth were influenced more by C, N, and P content in the leaf and root than in the stem (Figure 5B). And the correlation between C, N, and P contents and ratios in plant organs and soil depths were significantly higher at 10–30 and 30–60 cm depths than at 0–10 and 60–100 cm depths.

**Figure 6:**
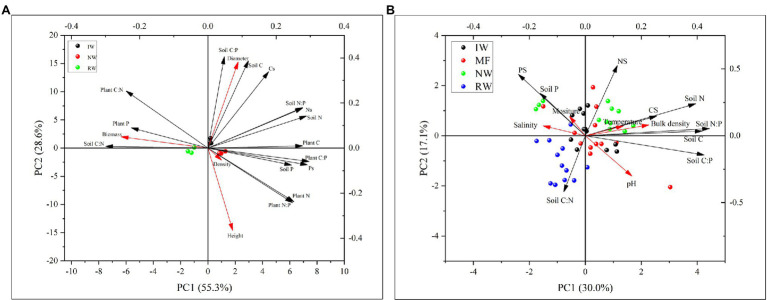
Principal component analysis (PCA) between plant growth traits and the C, N, P stoichiometry and stocks of plants and soils in three land-use types **(A)**. PCA analysis between soil physiochemical properties and the C, N, P stoichiometry and stocks of plants and soils in four land-use types **(B)**. MF, mudflat; NW, native *Phragmites australis*-dominated community wetland; IW, invasive *Spartina alterniflora*-dominated community wetland; and RW, reclaimed *P. australis*-dominated community wetland.

The PCA results revealed that the first two axes explained 83.9 and 47.1%, respectively, plant and soil C, N, and P contents and ratios were mainly affected by tidal wetlands (Figure 6). Plant and soil C, N, and P contents and ratios were closely related to plant growth traits (e.g., aboveground biomass and diameter; Figure 6A) and soil physiochemical properties (e.g., bulk density, temperature, and moisture; Figure 6B).

**Figure 7:**
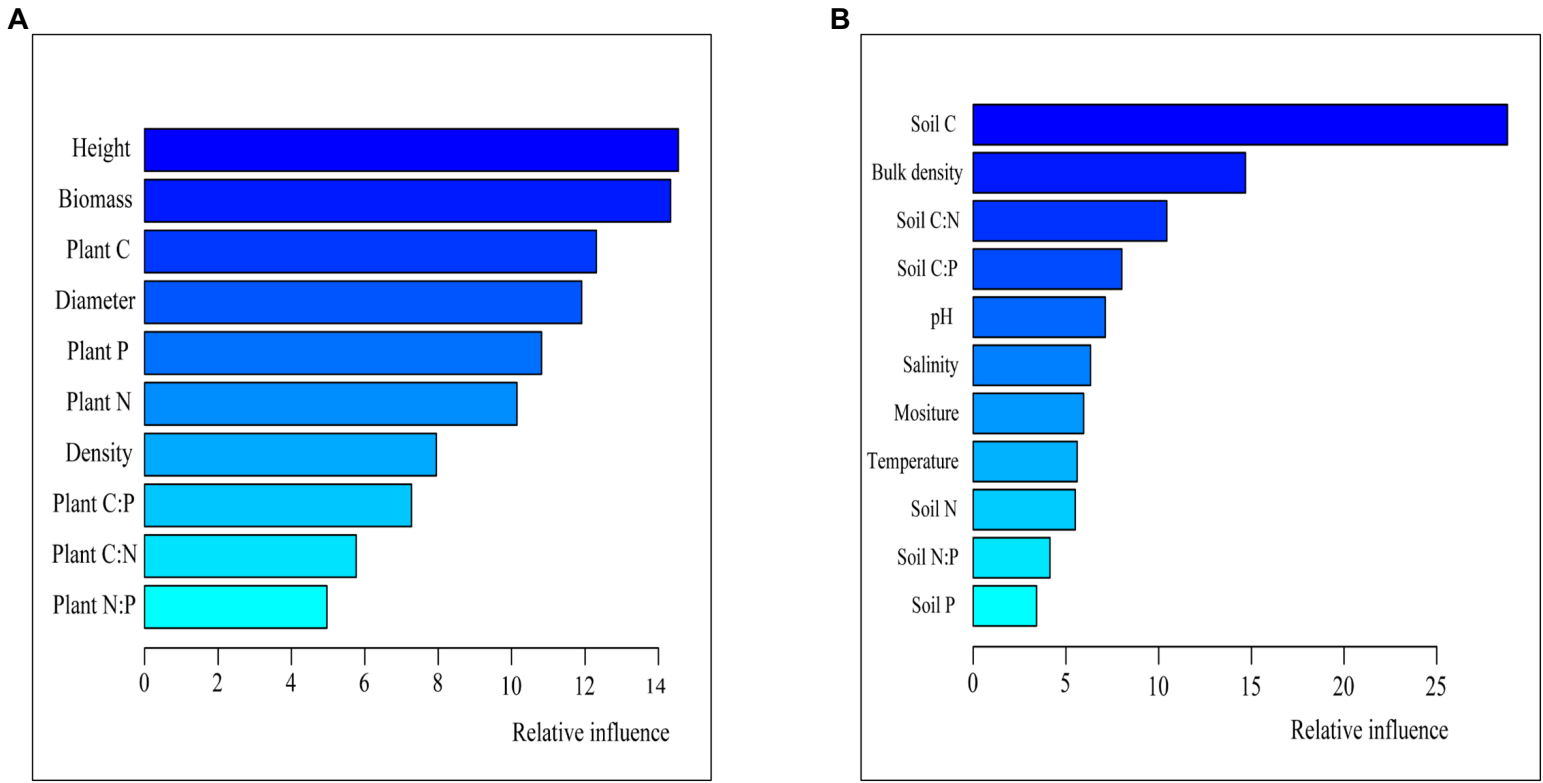
Aggregated boosted tree (ABT) analysis of the soil C, N, and P stocks, plant growth traits, and C–N–P stoichiometry **(A)**. ABT analysis of the soil C, N, and P stocks, soil physiochemical properties, and C–N–P stoichiometries **(B)**.

Soil Cs and Ns were more likely to be correlated with C, N, and P contents and ratios in soil than in plants, while soil Ps were both affected (Figure 5A). The effects of plant growth traits, especially the height and aboveground biomass, were higher than plant stoichiometry (Figure 7A), and the contributions of the soil C content and bulk density were greater than those of the other soil characteristics (Figure 7B).

**Figure 8:**
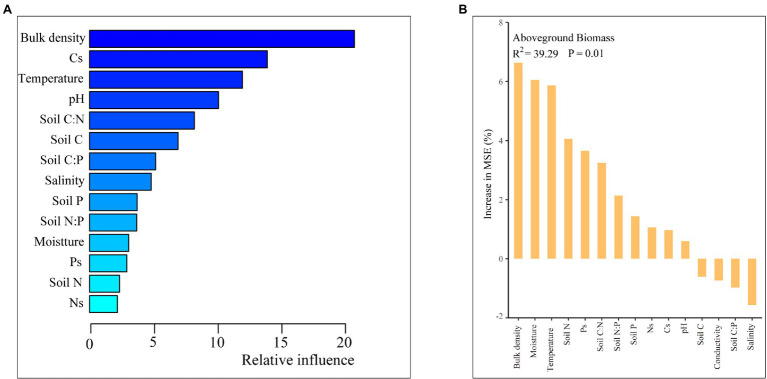
Aggregated boosted tree (ABT) analysis **(A)** and random forest analysis of aboveground plant biomass and soil properties **(B)**.

The plant growth traits were closely related to plant N and P contents and N:P ratio, and soil C:N and C:P ratios (Figure 6A). Except for plant and soil stoichiometry, the ABT analysis indicated that the plant growth traits were greatly affected by soil properties such as soil bulk density (Figure 8A). And the random forest analysis results showed that the aboveground biomass was mainly affected by soil physiochemical properties than soil C, N, and P contents and ratios and stocks (Figure 8B).

## Discussion

### Response of C, N, and P Contents and Allocation Patterns of Plant Organs to Land-Use Types

Coastal wetland can provide many special ecosystem services and products. The changes to Chinese coastal wetlands in the last few decades, such as land-use changes due to human activities, are especially important and universal (Ma et al., 2014; Wang et al., 2014a). Coupled biogeochemical cycles of C, N, and P are fundamental for primary production and organic matter accumulation/decomposition in coastal wetland (Hu et al., 2018, 2020; Wang et al., 2019). Plant nutrient content depends on the dynamic balance between plant demand and soil nutrient supply (Hu et al., 2020). Our results suggest that land-use has an important effect on plant C, N, and P contents and patterns. Compared to the NW, plant C and N of the RW and IW were lower. Firstly, significantly lower soil N contents and stocks in the RW may result in significantly lower plant N content (Chen and Chen, 2021). There was no significant difference in soil N content between the NW and IW; however, as the IW is in a low-tide area, denitrification caused by long-term flooding may reduce the availability of nitrate N, thus significantly reducing their plant N content (Ordoñez et al., 2010). Additionally, according to the growth dilution theory (Townsend et al., 2007), plant nutrient concentration will be diluted with the acceleration of the rapidly increasing biomass and decrease in nutrient content. The higher aboveground biomass in the RW and IW suggests their dilution effects may be stronger than the NW and may lower their C and N contents of plant organs.

The allocation patterns of C, N, and P in plant organs are limited by soil nutrient conditions (He et al., 2015; Ma et al., 2019) and are related to tissue structure and functional differentiation (Minden and Kleyer, 2014). During the plant life story, the plant can regulate element allocation in organs to adapt to the living environment and ensure high productivity (Zhang et al., 2020). Leaves have important functions of C assimilation and nutrient gathering, and their nutrient content is generally higher than that of root and stem, and not affected by exogenous nutrient additions (Hu et al., 2014; Xiong et al., 2021). Similarly, P content was significantly higher in leaf than in root and stem, which is conducive to synthesizing organic matter and promoting of plant growth and defense system (Wang et al., 2019). However, C and N contents were only significantly higher in leaf than in root and stem in the NW, while there were significantly lower in the RW or were no significant differences in the IW. The higher aboveground biomass may be the main reason leaf C and N contents were smaller than root and stem (Townsend et al., 2007). In addition, when plant growth is limited by nutrients, the nutrients are higher in root than in other organs (Graciano et al., 2005). The higher N allocation in root in the RW and IW will benefit root biomass, and then obtain more N nutrients absorbed.

### Response of Soil C, N, and P Contents and Stocks to Land-Use Types

Soil C, N, and P contents and profile patterns are closely related to their sources. Soil C and N contents decrease with soil depth and mainly originate from the input of plant litter and root and microbial residues (Yu and Chi, 2019; Zhang et al., 2019). Soil P content is more derived from soil rock weathering and has stable soil profile patterns (Vitousek et al., 2010). Similarly, soil P did not show distinct soil profile patterns, and soil C and N content decreased from 0 to 60 cm. In contrast, soil C and N contents at the 60–100 cm depth were more variable in the four different wetlands, mainly related to whether the root could reach the deep soil and the historic organic carbon accumulation. The soil profile patterns of Cs were similar to soil C content in four land-use types, while soil Ns and Ps were increased at a depth of 100 cm. Soil nutrient stocks were more closely related to soil properties and plant growth traits, indicating that land-use affected soil nutrient stocks by changing soil properties especially the soil bulk density (Wang et al., 2014a, 2015) and plant input (Yu and Chi, 2019). In addition, soil C, N, and P contents and stocks were more affected by tide, and soil total Cs, Ns, and Ps were similar in tidal wetland. Compared to tidal wetlands, soil C, N, and P content and stocks in the RW reached a minimum, indicating that high soil nutrient stocks were mainly attributed to the material exchange by tides (Peñuelas et al., 2012), and that reclamation was detrimental to nutrient storage.

### Connection Between Plant and Soil C:N:P Ratios

Soil C:N:P ratios are highly susceptible to human activities and climate factors (Peñuelas et al., 2012; Bui and Henderson, 2013; Yu et al., 2018) and further affect plant stoichiometry (Hu et al., 2020). Demars and Edwards (2010) showed that even under high nutrient supply conditions, the N:P ratios in wild plant tissues of 41 wetland plant species changed only slightly, whereby a strict N:P ratio was determined by the plant endostatic mechanism (Sterner and Elser, 2002). In this study, the N:P ratio pattern of plant organs was not affected by land-use type, indicating that the pattern of N:P ratio in plant organs had a certain internal stability. However, plant N:P was significantly lower after alien plant invasion or wetland reclamation, indicating that land-use types could significantly change the N:P ratio balance in coastal wetland ecosystem, which may change the status of plant nutrient limitation. Given that leaf N:P ratio could be an indicator of nutrient limitation of vegetation (Koerselman and Meuleman, 1996; Vitousek et al., 1996; Güsewell et al., 2003; Wang and Moore, 2014b). Therefore, plants in the NW (10/14 < leaf N:P ratio < 16/20) were co-limited by N and P, plants in the RW and IW (leaf N:P ratio < 10/14) were limited by N in this study.

However, many factors affect leaf N:P ratio. First, wetland herbs have a higher relative growth rate than terrestrial plants (Agren, 2004), which leads to a significant decrease in leaf N content and reducing leaf N:P ratio (Sardans et al., 2012). Second, plant nutrient content is related to soil nutrient availability rather than content (Koerselman and Meuleman, 1996; Cordell et al., 2001). The soil C:N:P ratio greatly determine nutrient availability for plants and soil microorganisms (Vitousek et al., 1996). Stronger correlations were found between the C, N, and P contents and ratios of plant organs and soil depths of 10–30 and 30–60 cm than soil depths of 0–10 and 60–100 cm, suggesting that plant nutrient content was mainly related to soil nutrient availability of 10–30 and 30–60 cm soil depths. At the 10–30 and 30–60 cm soil depths, significant lower soil C:N ratios and higher soil C:P and N:P ratios in NW means higher N and lower P availability as concluded earlier (Don et al., 2007; Iost et al., 2007; Wang et al., 2014a; Bing et al., 2016). Similarly, in the RW and IW, significantly higher soil C:N ratio and lower soil C:P and N:P ratios indicated lower N and higher P availability. When the availability of soil N and P were relatively low and high, respectively, the plant N:P ratio will be low (Güsewell, 2004). Compared to coastal tidal wetlands in eastern China (leaf N:P ratio was 7.55; Hu et al., 2018), and global coastal wetlands (leaf N:P ratio was 13.40; Hu et al., 2020), leaf N:P ratio of the NW was larger (leaf N:P was 15.33), and leaf N:P ratio of the RW and IW were smaller (leaf N:P were 3.00 and 5.13 respectively). Plant growth in the NW was co-limited by N and P nutrient, while plants in the RW and IW were limited by N nutrient.

### The Plant Growth Strategy of Different Land-Use Types

Carbon, N, and P contents and ratios of the plant are helpful in understanding plant growth strategy and its adaptability to environmental changes and stressors, and further contribute to ecological conservation and environmental protection (Elser et al., 2000; Vrede et al., 2004; Hessen et al., 2007; Rong et al., 2015; Zheng et al., 2021). Our results indicated that land-use may significantly alter the balanced C, N, and P contents and ratios in wetland ecosystems and significantly influence plant growth strategy. Moreover, results expressed that plant growth traits were closely related to plant N:P ratio, and soil C:N and C:P ratios. According to the growth rate hypothesis (Sterner and Elser, 2002), fast-growing species tend to have higher plant P content and lower plant C:P and N:P ratios than slow-growing species. In the NW with lower soil P availability and higher N availability, the significant lowest plant P content and highest plant C:P and N:P ratios indicated that plants tend to adopt a conservative resource acquisition strategy, which may lead to significant lowest aboveground biomass. In the RW with lower soil N availability and higher P availability, the significant highest plant P content and lowest plant C:P and N:P ratios indicated that plants tended to adopt a rapid resource acquisition strategy, so its aboveground biomass reached a significant highest value. In the IW, plant P content, C:N and C:P ratios, soil N and P availability, and aboveground biomass were between those of the NW and RW. These findings suggest that the plant growth strategy of the IW was also between that of the NW and RW. However, C, N, and P contents and ratios of plants and soils are all affected by soil physiochemical properties. In addition, plant growth traits were not only affected by soil nutrient limitation and availability, but also by soil physicochemical properties such as bulk density. Therefore, land-use may affect stoichiometry by altering soil physiochemical properties, and then affect vegetation growth strategy.

## Conclusion

Land-use in Hangzhou Bay coastal wetland affected the C, N, and P contents and ratios of plant and soil by changing the soil’s physiochemical properties, thus affecting the nutrient availability and stocks, and eventually affecting plant growth. In tidal wetlands, the difference in soil C, N, and P stocks was not significant, while its N and P availability varied. Compared to the NW, soil P availability of IW was higher, and N availability was lower. Compared to tidal wetland, N stocks and availability of the RW were smaller, while its soil P availability was higher. Moreover, changes in soil C, N, and P stocks and availability ultimately lead to plants taking different growth strategies. In the NW, plants were co-limited by N and P nutrients and took a conservative growth strategy. In the RW, plants were limited by N nutrient and took a rapid growth strategy. In the IW, plants were limited by N nutrient and took a slow-rapid growth strategy. In conclusion, both plant growth and soil nutrient status are closely related to land-use. Reclamation and plant invasion are beneficial to vegetation growth at present, while severe N-limitation and smaller N and P stocks are not beneficial to vegetation community development in the long term. Additionally, the IW is located at the low-tide zone, the lower N availability has negative consequences for water quality since it promotes eutrophication processes. The soil nutrient management, especially N fertilizer, and dynamic monitoring of water quality of different land-use in coastal wetland should be strengthened in the future.

## Data Availability Statement

The raw data supporting the conclusions of this article will be made available by the authors, without undue reservation.

## Author Contributions

All authors contributed to the study and manuscript preparation. XS and MW are responsible for ensuring that the descriptions are accurate and agreed upon by all the authors. JX: conceptualization, methodology, software, data curation, writing—original draft, writing—review and editing, and visualization. XS and MW: conceptualization, methodology, writing—review and editing, validation, project administration, and funding acquisition. HY and EL: methodology, investigation, and resources. All authors contributed to the article and approved the submitted version.

## Funding

This study was financially supported by the National Natural Science Foundation of China (31870597), the Special Fund for Cooperation of Zhejiang Province, and the Chinese Academy of Forestry (2021SY03).

## Conflict of Interest

The authors declare that the research was conducted in the absence of any commercial or financial relationships that could be construed as a potential conflict of interest.

The handling editor declared a shared affiliation with the authors at the time of review.

## Publisher’s Note

All claims expressed in this article are solely those of the authors and do not necessarily represent those of their affiliated organizations, or those of the publisher, the editors and the reviewers. Any product that may be evaluated in this article, or claim that may be made by its manufacturer, is not guaranteed or endorsed by the publisher.
