# Supplementary material for: Carbon, Nitrogen, and Phosphorus Stoichiometry and Plant Growth Strategy as Related to Land-Use in Hangzhou Bay Coastal Wetland, China
Source: Front Plant Sci. 2022 Jul 6;13:946949. doi: 10.3389/fpls.2022.946949 (PMC9298656; doi:10.3389/fpls.2022.946949)
Supplement: Supplementary file 1 [file Data_Sheet_1.docx]

Supplementary Material

# Supplementary Figures and Tables

## Supplementary Figures


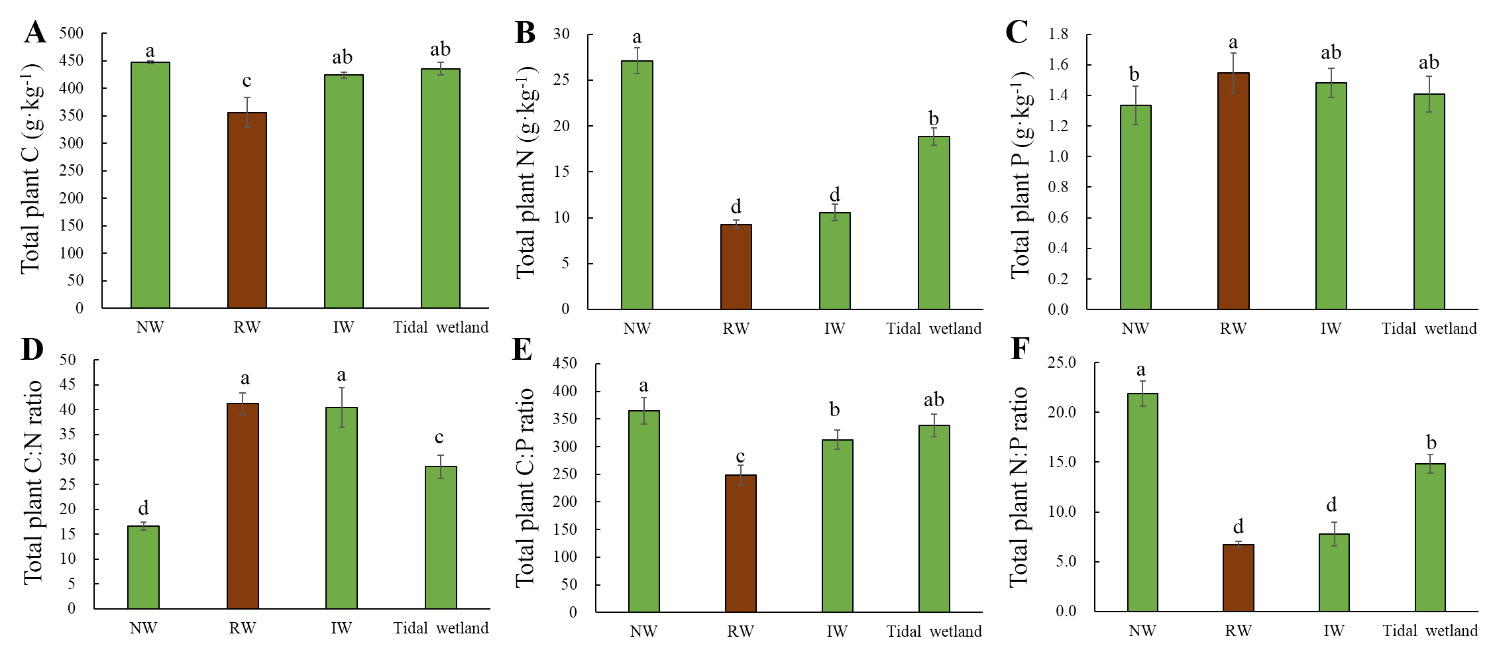


## Figure S1. Distributions of total plant C contents (A), N content (B), P content (C) and C:N ratio (D), C:P ratio (E), N:P ratio (F) (means ± standard deviation, n = 3) in different land-use types in Hangzhou Bay. Different lower-case letters indicate a significant difference among land-use types at *P* < 0.05.

**
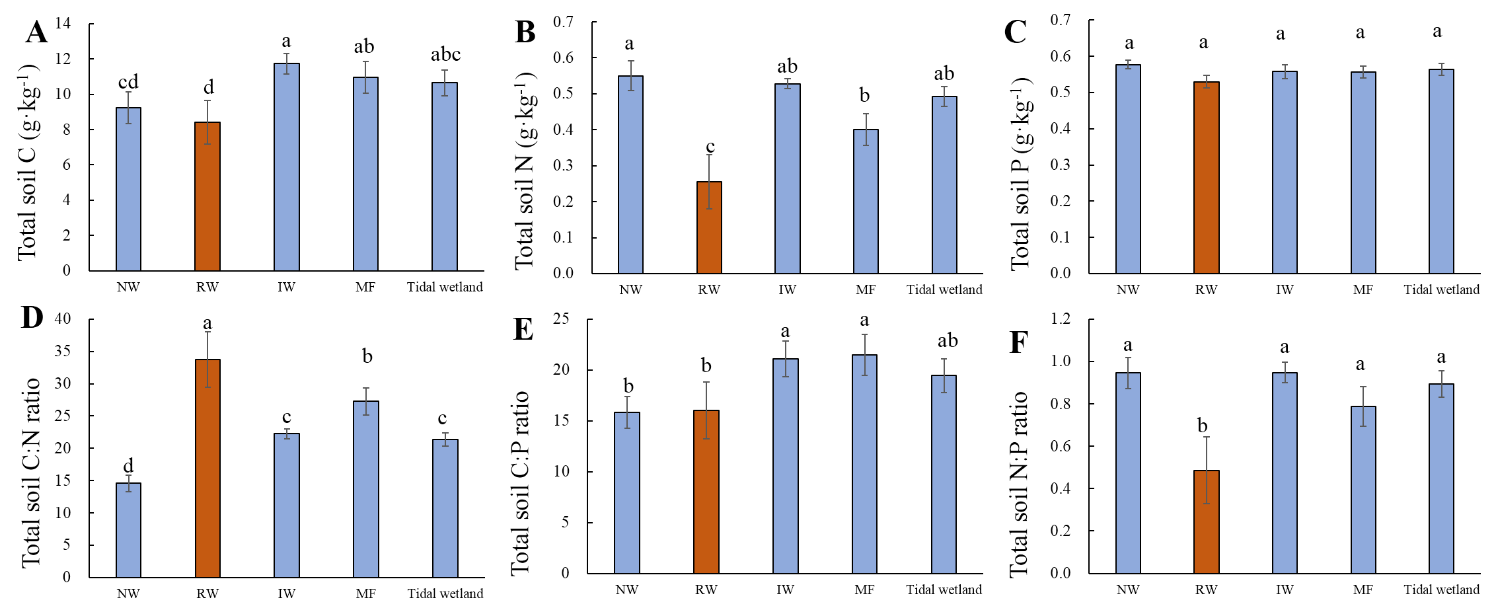
**

## Figure S2. Distributions of total soil C contents (A), N content (B), P content (C) and C:N ratio (D), C:P ratio (E), N:P ratio (F) (means ± standard deviation, n = 3) in different land-use types in Hangzhou Bay. Different lower-case letters for soil indicate a significant difference among land-use types at *P* < 0.05.

## 1.2 Supplementary Tables

**Table S1.** Plant growth traits of different land-use types in Hangzhou Bay

| Wetland type | Diameter  (cm) | Height  (cm) | Density  (per plants·m^-2^) | Aboveground biomass  (g·m^-2^) |
| --- | --- | --- | --- | --- |
| MF | — | — | — | — |
| NW | 9.95 ± 0.60bc | 324.80 ± 12.18a | 85.67 ± 16.50a | 1469.35 ± 477.59c |
| RW | 9.11 ± 0.82c | 262.60 ± 23.81b | 84.67 ± 2.89a | 3261.70 ± 375.90a |
| IW | 12.96 ± 1.50a | 150.67 ± 19.13c | 84.00 ± 12.17a | 2293.67 ± 512.69b |

Different lowercase letters indicate significant differences among land-use types at *P* < 0.05.

**Table S2.** Table S2 Soil physiochemical properties of different land-use types in Hangzhou Bay

| Wetland type | Soil depth (cm) | Temperature (°C) | Moisture (%) | pH | Salinity (g·kg^-1^) | Bulk density (g·cm^-3^) |
| --- | --- | --- | --- | --- | --- | --- |
| MF | 0–10 | 28.17 ± 1.02Aa | 49 ± 15Aa | 9.44 ± 0.17Aa | 3.76 ± 0.45Aa | 1.33 ± 0.10Aa |
|  | 10–30 | 26.37 ± 0.78Bab | 41 ± 2Ab | 9.40 ± 0.22Aa | 4.34 ± 1.16Aa | 1.25 ± 0.05Aa |
|  | 30–60 | 25.37 ± 1.10ABab | 39 ± 1Aab | 9.66 ± 0.20Aa | 3.77 ± 0.99Aa | 1.17 ± 0.09Aa |
|  | 60–100 | 24.13 ± 0.40Cb | 41 ± 3Aab | 9.51 ± 0.23Aa | 3.83 ± 0.85Aa | 1.25 ± 0.01Aa |
| NW | 0–10 | 26.67 ± 0.12Ab | 48 ± 5Aa | 9.27 ± 0.39a | 3.43 ± 0.67Ba | 1.28 ± 0.14Aa |
|  | 10–30 | 26.13 ± 0.15Ab | 48 ± 4Aab | 8.96 ± 0.21Aab | 3.96 ± 0.57Aa | 1.08 ± 0.13Aab |
|  | 30–60 | 24.70 ± 0.53Bb | 42 ± 2Aab | 9.02 ± 0.11Aab | 3.82 ± 0.37Aa | 1.10 ± 0.03Aab |
|  | 60–100 | 23.70 ± 0.10Cb | 45 ± 6Aab | 8.78 ± 0.26Ab | 4.75 ± 0.51Aa | 1.16 ± 0.14Aab |
| RW | 0–10 | 27.03 ± 0.06Ab | 29 ±4Ab | 9.53 ± 0.39Aa | 4.15 ± 2.80Aa | 1.21 ± 0.21Aab |
|  | 10–30 | 26.93 ± 0.06Aa | 31 ±3Ac | 9.63 ± 0.62Aa | 4.74 ± 3.11Aa | 1.01 ± 0.12Aa |
|  | 30–60 | 26.27 ± 0.47Ba | 38 ±8Ab | 9.44 ± 0.69Aa | 5.72 ± 3.45Aa | 0.95 ± 0.06Ab |
|  | 60–100 | 24.70 ± 0.17Ca | 36 ±5Ab | 9.31 ± 0.58Aab | 5.80 ± 3.18Aa | 0.99 ± 0.0.03Ac |
| IW | 0–10 | 27.50 ± 0.26Aab | 50 ± 7Aa | 8.94 ± 0.51Aa | 4.74 ± 2.28Aa | 1.16 ± 0.10Aa |
|  | 10–30 | 25.70 ± 0.10Bb | 51 ± 5Aa | 8.63 ± 0.28Ab | 5.83 ± 1.11Aa | 1.03 ± 0.10Ab |
|  | 30–60 | 24.57 ± 0.50Cb | 49 ± 7Aa | 8.64 ± 0.22Ab | 5.39 ± 0.53Aa | 1.02 ± 0.08Abc |
|  | 60–100 | 23.70 ± 0.17Db | 48 ± 2Aa | 8.92 ± 0.17Aab | 5.22 ± 0.71Aa | 1.06 ± 0.06Abc |

Different capital letters in the same land-use type indicate a significant difference among soil depths at *P* < 0.05, and different lowercase letters in the same soil depth indicate a significant difference among land-use types at *P* < 0.05.
